# The quest for the ideal anato-molecular imaging fusion tool

**DOI:** 10.2349/biij.2.4.e47

**Published:** 2006-10-01

**Authors:** H Zaidi

**Affiliations:** Geneva University Hospital, Division of Nuclear Medicine, CH-1211 Geneva, Switzerland

The past decade has witnessed major scientific and technological advancements, and one among them was molecular imaging. Today, molecular imaging constitutes a major trend in biomedical research and seems to have the potential to revolutionise life sciences. Molecular imaging plays a valuable role in the assessment of cellular targets and the response to therapy, differential diagnosis, prediction or selection of patients who will benefit from treatment, and in dosimetry for targeted therapy. The field of preclinical and clinical molecular imaging has developed simultaneously with molecular medicine, which holds great promise to provide significant healthcare benefits in the future. The advent of dual-modality PET/CT units is a prominent example of advance in molecular imaging technology. It offers the opportunity to modernise the practice of clinical oncology by improving lesion localisation and facilitating treatment planning for radiation therapy.

Although dual-modality imaging systems designed specifically for clinical practice are a recent feature, the potential advantages of combining anatomical and functional imaging has been recognised for several decades by radiological scientists and physicians [1]. Many of the pioneers of nuclear medicine recognised that a radionuclide imaging system could be augmented by adding an external radioisotope source. This would acquire transmission data for anatomical correlation of the emission image. The conceptual designs were, however, never introduced in practice or implemented in either an experimental or a clinical setting until Hasegawa and colleagues (University of California, San Francisco) pioneered in the 1990s the development of dedicated SPECT/CT [2,3]. Later, Townsend and co-workers (University of Pittsburgh) pioneered in 1998, the development of combined PET/CT imaging systems. These have the capability to record both PET emission and x-ray CT data for correlated functional/structural imaging [4,5]. Thereafter, PET/CT dual-modality imaging systems were introduced by the major scanner manufacturers for routine clinical use. According to market reports, over 90% of today’s PET sales are combined PET/CT units. This led almost all scanner manufacturers to opt for replacing entirely PET-only scanners by PET/CT. While all clinical and commercial dual-modality systems have been configured in the form of PET/CT or SPECT/CT scanners, several investigators proposed, implemented, and tested prototype combined PET/MRI imaging systems [6]. PET/MRI is a more challenging technology compared with PET/CT. The importance of this development will only be understood and manifest when this and other forms of dual-modality imaging become available in the ensuing years and are utilised for clinical studies of humans as well as biological investigations involving animal models of human disease.

Since its inception, PET/CT has been advertised as a cutting-edge technology to influence clinicians and decision makers to adopt it as the new gold standard modality and to push scanner manufacturers to replace standalone PET scanners with combined PET/CT units. The latter is considered a questionable choice by some of the pioneers in this field, with whom the author concurs [7]. The marketing strategy of vendors supported by many scientists aiming at disseminating PET/CT technology in the clinic is that the added value of combined units is well-established and represents the ultimate solution for image co-registration. According to them, this solution enables appropriate combination of imaging technologies to yield useful anato-molecular imaging fusion [8]. The bottom line is that although PET/CT has been accepted commercially, the clinical benefits and the need for this technology remains controversial [9,10]. These issues are still being debated [7,11]. While hybrid PET/CT has many interesting features and offers several advantages compared with software approaches of image co-registration for patient diagnosis and image-guided radiation therapy, it is often argued that combined PET/CT is not the ultimate solution for image co-registration [12,13]. It is also possibly not considered a major breakthrough that revolutionised the paradigm of medical imaging [11].

The use of the noise-free CT data for attenuation correction of PET images has indisputably several virtues compared with conventional radionuclide-based transmission scanning. It should, however, be recognised that its clinical benefits have not been unequivocally demonstrated and should be carefully documented by investigators for wider acceptance. The key point is that many PET procedures do not require a diagnostic quality CT, and radionuclide-based transmission scanning would be a better option than low-dose CT protocols. It is still too early to claim that transmission scanning devices are obsolete for PET/CT, and that CT-based attenuation correction should be the gold standard on these systems [14]. In my opinion, transmission scanning has a genuine role and remains an appealing alternative until all the problems associated with CT-based attenuation correction are resolved through research [15].

It is the responsibility of clinical scientists and medical physicists providing support to clinical PET facilities and involved in today’s biomedical imaging research enterprise to debate on important issues about the introduction of new technologies. They must educate and advise clinical end users who often make choices under the influence of advertisements and the pressure of competitors. Any new technology should be assessed carefully with respect to benefits conveyed to patients. I share the opinion of the same pioneer mentioned above [7] and emphasise that we clearly need a worldwide debate involving all potential users of this technology on how best to adapt to novel information and technological progress. We urgently need large-scale studies to demonstrate the clinical benefits of PET/CT and, more importantly, to define where PET alone is needed and where PET/CT is needed.

PET/CT is poised to advance the application of molecular diagnosis in oncology, neurology, cardiology, infectious diseases, and other types of disease. Nevertheless, PET/CT is obviously not the only major non-invasive tool for the assessment of human disease. New technologies, such as, high-field MRI and bioluminescent and fluorescent imaging have blurred the artificial distinction that, in the past, set nuclear medicine as a “functional” rather than “anatomic” imaging modality [16]. PET/CT definitely maintains an exclusive standing in the delivery of targeted therapies, but its superior picomolar sensitivity is being challenged by competing technologies, such as those using ultra small superparamagnetic contrast agents [17].
